# Molecular Docking and Energetic Analysis of Deferoxamine in Uropathogenic *Escherichia coli* in an Experimental Model

**DOI:** 10.3390/microorganisms14071590

**Published:** 2026-07-21

**Authors:** Mayane Cristina Pereira Marques, Flávia Danyelle Oliveira Nunes, Camila Evangelista Carnib Nascimento, José Lima Pereira-Filho, Israel Viegas Moreira, Ana Beatriz Santos Sousa, Aline Santana Figueredo, Roseane Lustosa de Santana Lima, Gabriel Moreira Pereira, Raysa Lins Caldas, Antônio Silva Machado, Rosilda Silva Dias, Jaiza Sousa Penha, Bruna Caroline Silva Falcão, Phelipe Austríaco Teixeira, Marliete Carvalho da Costa, Joicy Cortez de Sá Sousa, Caio Pavão Tavares, Valério Monteiro-Neto, Eduardo Martins de Sousa, Rafael Cardoso Carvalho

**Affiliations:** 1 Graduate Program in Health Sciences, Federal University of Maranhão (UFMA), São Luís 65080-805, Brazil; mayane.marques@discente.ufma.br (M.C.P.M.); flavia.danyelle@ufma.br (F.D.O.N.); camila.carnib@ufma.br (C.E.C.N.); jlp.filho@discente.ufma.br (J.L.P.-F.); israel.moreira@ufma.br (I.V.M.); ana.beatriz9@discente.ufma.br (A.B.S.S.); aline.sf@discente.ufma.br (A.S.F.); roseane.lustosa@discente.ufma.br (R.L.d.S.L.); gm.pereira@discente.ufma.br (G.M.P.); raysa.caldas@discente.ufma.br (R.L.C.); joicy.sa@ufma.br (J.C.d.S.S.); caio.pavao@ufma.br (C.P.T.); valerio.monteiro@ufma.br (V.M.-N.); eduardo.martins@ceuma.br (E.M.d.S.); 2 Medical School, CEUMA University (UNICEUMA), São Luís 65075-120, Brazil; neto.maxado@gmail.com; 3 Graduate Program in Nursing, Federal University of Maranhão (UFMA), São Luís 65080-805, Brazil; rs.dias@ufma.br (R.S.D.); jaiza.sousa@ufma.br (J.S.P.); 4 Graduate Program in Public Health, Federal University of Maranhão (UFMA), São Luís 65080-805, Brazil; bruna.falcao@discente.ufma.br; 5 Medical School, Health Sciences Center, State University of the Tocantina Region in Maranhão (UEMASUL), Imperatriz 65900-000, Brazil; phelipe.teixeira@uemasul.edu.br; 6 Graduate Program in Biosciences Applied to Health, CEUMA University (UNICEUMA), São Luís 65075-120, Brazil; cmarliete@yahoo.com

**Keywords:** neurogenic cystitis, urinary tract infection, iron-chelating agent, in silico analyses, siderophore

## Abstract

Neurogenic bladder is a condition associated with impaired voiding, leading to urinary stasis and increased susceptibility to urinary tract infections, predominantly caused by *Escherichia coli*. In this context, bacterial iron acquisition systems represent attractive targets for alternative antimicrobial strategies. This study aimed to investigate the interactions of deferoxamine with *E. coli* iron acquisition proteins, combining an experimental model of neurogenic bladder with molecular analyses. The experimental model of neurogenic bladder was induced by complete spinal cord transection in rats, followed by urine collection by cystocentesis and microbiological characterization of uropathogens. Subsequently, molecular docking and energetic analyses were performed to evaluate the binding of deferoxamine and its Fe-DFO complex to the FhuE receptor of the ferric hydroxamate uptake pathway, with FhuA and FhuD prepared as correlated targets of the same pathway for structural context. The animals presented urinary retention and bacterial colonization, with *E. coli* identified as the pathogen. The results of the molecular docking revealed geometrically plausible accommodation of Fe-DFO within siderophore recognition pockets, involving residues associated with siderophore recognition and transport, as well as binding affinity scores consistent with weak-to-moderate structural complementarity compared to reference ligands. It is concluded that the neurogenic bladder model provides a biologically relevant framework for the study of urinary tract infections and that deferoxamine exhibits molecular interactions consistent with the ferric hydroxamate uptake system of *E. coli*. Because the present analysis was restricted to the Fhu pathway, these findings cannot be extrapolated to overall bacterial iron homeostasis, which involves multiple parallel acquisition systems. The current work is explicitly positioned as a proof-of-concept investigation; in vivo administration of DFO in the neurogenic bladder model, functional assays of iron uptake, transporter specificity experiments, and molecular dynamics analyses are identified as priority directions for future work.

## 1. Introduction

Neurogenic bladder (NB) results from lesions of the nervous system that alter bladder innervation, impairing urinary storage and voiding. This condition leads to dysfunctions such as urinary urgency, incontinence, increased post-void residual volume, and a high risk of pyelonephritis and renal damage [1,2]. These conditions favor urinary tract infections (UTIs) due to disruption of the urothelial barrier, impairment of local immunity, urinary stasis, and the frequent use of catheterization. Some bacteria are particularly favored in this environment, such as *Pseudomonas*, *Proteus*, *Escherichia coli*, and *Enterococcus* [3].

Playing central roles in key microbial metabolic processes, such as respiration, DNA synthesis, and redox metabolism, iron is an essential micronutrient. However, under physiological conditions, the availability of ferric iron (Fe^3+^) is extremely low due to its poor solubility and the host’s ability to sequester this metal through proteins such as transferrin and lactoferrin, thereby limiting its availability to invading microorganisms. This limitation imposes a significant nutritional challenge for bacterial pathogens, which depend on iron for multiplication and virulence [4].

To overcome iron scarcity, Gram-negative bacteria such as *Escherichia coli* have developed highly specialized iron acquisition systems based on the synthesis and utilization of siderophores—small, low-molecular-weight molecules with extremely high affinity for Fe^3+^. Once secreted, these siderophores form complexes with Fe^3+^ in the extracellular environment, which are then taken up by specific receptors in the outer membrane, such as TonB-dependent proteins, and transported into the cell by ABC transport systems, releasing iron for metabolic utilization [5,6].

In addition to their nutritional function, siderophores play important roles in bacterial pathogenicity. They not only enable iron acquisition in restrictive environments but are also considered critical virulence factors in bacterial infections, since the ability to efficiently compete for iron against host defense mechanisms is a determining factor for infectious success [7]. Emerging therapeutic strategies have precisely explored this bacterial dependence on iron, aiming to deprive the pathogen of its metal source or to block acquisition pathways, as an alternative or adjunct approach to classical antimicrobial therapies, especially in view of the increasing resistance to conventional antibiotics [8,9].

Deferoxamine (DFO) is a hydroxamate-type iron-chelating agent clinically used for the treatment of iron overload in diseases such as thalassemia. Its chemical structure allows the formation of highly stable complexes with Fe^3+^ through three bidentate hydroxamate groups. Although its direct use as an antimicrobial agent has shown limited efficacy in some microbiological contexts, DFO remains a relevant prototype for investigating iron-limitation mechanisms and their impact on bacterial growth [10]. In particular, the recent literature suggests that metabolic modulation approaches associated with iron availability may reveal exploitable vulnerabilities in resistant pathogens [11].

Despite the existing knowledge about the molecular mechanisms of iron acquisition by natural siderophores, there remains a gap in the detailed understanding of the interactions between exogenous chelators such as DFO and *E. coli* bacterial proteins at the structural and energetic levels. Computational tools such as molecular docking and binding energy analyses have proven to be effective in predicting binding modes and evaluating the feasibility of interactions between ligands and target proteins, contributing to the identification of patterns that may guide new therapeutic strategies or the modification of existing compounds.

The neurogenic bladder model induced by spinal cord injury is a well-established platform for the study of urinary tract infection; prior work has characterized the enhanced susceptibility to infection, exaggerated inflammatory response, and delayed bacterial clearance that occur in spinal cord-injured hosts, and has shown that these outcomes are governed by host-dependent mechanisms not determined solely by urinary stasis [12]. In the present study, this model is therefore employed not to generate novel microbiological findings, but to provide a clinically relevant and biologically validated infection context that anchors the selection of bacterial iron acquisition targets for computational analysis. In this context, the present study employs molecular docking and energetic characterization to investigate the interactions of deferoxamine, both in its free form and complexed with iron, with the FhuE receptor of the ferric hydroxamate uptake pathway of *E. coli*, aiming to provide a structural and energetic basis for hypothesis-driven future investigation of DFO as a candidate for iron acquisition interference studies in uropathogenic bacteria.

## 2. Materials and Methods

### 2.1. Experimental Surgical Model

The experimental model of neurogenic bladder was induced by complete spinal cord transection in adult *Rattus norvegicus* (Wistar strain) rats, 60 days old, of both sexes, totaling six animals (n = 6). The sample size was determined based on the high phenotypic reproducibility of the spinal cord transection model, in which complete loss of motor and bladder function is consistently achieved in all animals; this sample size is also consistent with published studies employing equivalent neurogenic bladder models in rodents for microbiological characterization purposes. The primary objective of the animal model component was to validate the infection context and to isolate a clinically relevant uropathogenic strain rather than to generate parametric comparative data requiring formal power analysis. The animals were obtained from the Animal Facility and Center for Experimental Research of the Federal University of Maranhão and were maintained under controlled vivarium conditions, including ambient temperature (24 ± 2 °C), humidity (50 ± 5%), and light–dark cycle (12 h/12 h), with free access to water and standard chow ad libitum until euthanasia, except for a two-hour food and water fast immediately prior to the experimental surgical procedure. Animals were acclimatized for 15 days before the surgical procedure in Multiuser Laboratory 2 of the Animal Facility and Center for Experimental Research (BCEA/UFMA). During the acclimatization period, environmental enrichment was provided in accordance with the facility’s standard protocols: structural refuge-type enrichment (Alesco, Monte Mor, SP, Brazil) during the first week, and physical/exploratory enrichment with disposable non-toxic kraft paper passage tubes (Relax, Granja RG, Suzano, SP, Brazil) during the second week. For the present experimental condition, animals were housed two per cage in crystal polycarbonate rat cages fitted with a low-profile wire-bar lid (Alesco, Monte Mor, SP, Brazil) to facilitate access to water and feed without requiring the animal to assume a bipedal position, thereby accommodating the postoperative motor deficit. All experimental procedures were conducted in accordance with the guidelines of the Brazilian Society of Laboratory Animal Science (SBCAL) and were approved by the Institutional Animal Care and Use Committee (CEUA/UFMA), under protocol no. 23115.002717/2023-07 (approved on 14 April 2023).

The anesthetic protocol consisted of the intraperitoneal administration of dexmedetomidine (50 mcg/kg), midazolam (5 mg/kg), and tramadol (10 mg/kg), with maintenance of the anesthetic plane via a face mask delivering 2.5% isoflurane using an inhalational anesthesia infusion system (Bonther Equipamentos & Tecnologia, Ribeirão Preto, SP, Brazil). Adequate anesthetic depth was confirmed by the absence of withdrawal and pedal reflexes prior to the start of the surgical procedure. Throughout the surgical procedure, heart rate and oxygen saturation (SpO_2_) were continuously monitored using a DL900 multiparameter monitor (Delta Life, São José dos Campos, SP, Brazil). To prevent intraoperative hypothermia, animals were maintained on a veterinary thermal mattress (Brasmed, Paulínia, SP, Brazil). After trichotomy of the dorsal region and antisepsis with 2% chlorhexidine solution, the animals were placed in the prone position. A longitudinal incision was performed in the thoracic region, followed by dissection of the epaxial musculature and subsequent laminectomy between the T12 and T13 vertebrae using a micro-rongeur.

During the postoperative period, animals received multimodal analgesia initiated immediately after the surgical procedure, comprising meloxicam (2 mg/kg, once daily) together with dipyrone (100 mg/kg) associated with tramadol (10 mg/kg), the latter combination administered every six hours until the time of euthanasia. During anesthetic recovery, body temperature and oxygen saturation were monitored every four hours, and animals were kept in a heating chamber with a red warming light and oxygen support delivered by face mask; body temperature was verified using a digital thermometer (G-Tech, Accumed-Glicomed, Duque de Caxias, RJ, Brazil). Postoperative monitoring for signs of pain was performed by the veterinarian of the BCEA/UFMA, the technical supervisor responsible for the study, using the Rat Grimace Scale (RGS) together with clinical and behavioral evaluation, in accordance with the guidelines of the Brazilian National Council for the Control of Animal Experimentation (CONCEA). The predefined humane endpoint for early intervention was the presence of signs of intense or unbearable pain; no animal reached this endpoint, and no unexpected adverse events occurred. The atonic bladder was managed 12 h after the surgical procedure by manual bladder massage (vesical expression) to promote bladder emptying. Twenty-four hours after the experimental procedure, functional evaluation was performed using the Basso, Beattie, and Bresnahan (BBB) locomotor rating scale. The Basso, Beattie, and Bresnahan (BBB) locomotor rating scale is a validated 22-point observational instrument (scores 0 to 21) developed for the assessment of hindlimb locomotor function following spinal cord injury in rats, where 0 indicates complete absence of observable movement and 21 corresponds to normal locomotion. BBB assessments were performed by two independent observers blinded to the surgical procedure, at 24 h post transection, in an open field arena under standardized conditions with each animal observed for a minimum of four minutes; inter-rater agreement was confirmed prior to data recording. Animals that presented scores between 0 and 1, indicating complete loss of voluntary hindlimb motor function and loss of micturition control, confirmed effective induction of the neurogenic bladder model. All six animals met this criterion, supporting the reproducibility of the model.

Subsequently, the animals underwent clinical evaluation, followed by urine collection for laboratory confirmation of cystitis. For this purpose, the animals were anesthetized with dexmedetomidine (50 mcg/kg) and midazolam (5 mg/kg) via intramuscular administration. After achieving an adequate anesthetic plane, under laminar flow conditions, the animals were placed in the supine position, followed by antisepsis of the abdominal and pelvic regions. They were then subjected to cystocentesis for urine collection by inserting a needle (0.6 mm) at a 45° angle into the retroperitoneal region, beneath the urinary bladder, to collect the urinary content (approximately 5 mL). The urine samples were subsequently sent for urine culture for isolation and identification of the microorganism causing cystitis, as well as for urinalysis (qualitative and quantitative analyses). All samples were processed within one hour of collection to prevent post-collection bacterial overgrowth. The selective and differential media employed (EMB agar, MacConkey agar, and chromogenic agar) provide inherent discrimination against commensal skin flora. Positivity was defined as bacterial counts equal to or greater than 10^5^ CFU/mL, consistent with true infection rather than contamination, which characteristically yields mixed flora at low counts. After sample collection, the animals were maintained under an anesthetic plane using a face mask delivering 2.5% isoflurane and were subsequently euthanized by terminal blood collection via the caudal vena cava.

### 2.2. Microorganism Identification

Isolation and phenotypic identification of *E. coli* were performed following the methodology described by Bernaitis et al. [13]. Briefly, samples were streaked onto Eosin Methylene Blue (EMB) agar plates and incubated at 37 °C for 24 h. In parallel, MacConkey agar and chromogenic agar were also used under the same incubation conditions to support selective isolation and preliminary differentiation of *E. coli* colonies.

Presumptive *E. coli* isolates were selected based on their typical colonial morphology on selective media and subsequently subjected to Gram staining. Smears were prepared, heat-fixed, and stained using the standard Gram staining protocol. Microscopic examination was performed to confirm the presence of Gram-negative bacilli, consistent with the morphological characteristics of *E. coli* [13].

### 2.3. Biochemical Characterization

Biochemical identification of the isolates was carried out according to the protocol described by Bernaitis et al. [13] using a panel of standard biochemical tests for enterobacteria. The catalase test was performed using 3% hydrogen peroxide, and the oxidase test was carried out using commercial oxidase discs. Indole production was evaluated in peptone water after incubation at 37 °C for 24 h using Kovac’s reagent. The methyl red (MR) and Voges–Proskauer (VP) tests were performed in MR-VP broth to assess mixed acid fermentation and acetoin production, respectively. Citrate utilization was tested on Simmons citrate agar, and urease activity was evaluated on Christensen’s urea agar slants.

Triple Sugar Iron (TSI) agar was used to assess glucose, lactose, and sucrose fermentation, as well as gas and hydrogen sulfide (H_2_S) production. In addition, carbohydrate fermentation tests were performed using individual sugars, with acid production detected by color change in the medium and gas production assessed using Durham tubes.

The biochemical profile obtained was interpreted according to standard criteria and compared with the reference identification scheme described by Bernaitis et al. [13] and Rojas et al. [14] to confirm the identification of *E. coli*.

### 2.4. Urinalysis

The physicochemical analysis of the urine was performed using commercial reagent test strips (Uriquest Plus I, Labtest Diagnóstica S.A., Lagoa Santa, MG, Brazil), according to the manufacturer’s instructions. The following parameters were evaluated: bilirubin, urobilinogen, ketone bodies, glucose, protein, blood, pH, nitrite, leukocytes, and specific gravity. Urine color, appearance, and odor were assessed by direct visual examination. For the evaluation of the urinary sediment, 5 mL aliquots of urine were transferred to conical tubes and centrifuged at 1500 rpm for 5 min. After centrifugation, the supernatant was carefully discarded, retaining approximately 0.5 mL for resuspension of the sediment, which was subsequently analyzed by optical microscopy to detect red blood cells (RBCs), white blood cells (WBCs), casts, and crystals.

### 2.5. Molecular Docking

#### 2.5.1. Ligand Acquisition and Preparation

The structure of the ligand deferoxamine (DFO) was obtained from the PubChem database (https://pubchem.ncbi.nlm.nih.gov) (CID: 2973) in 2D Structured Data File (.sdf) format.

#### 2.5.2. Protein Preparation

The structures of the *E. coli* outer membrane receptor FhuE (PDB ID: 6E4V, resolution: 2.00 Å) [13], the outer membrane receptor FhuA (PDB ID: 1BY5, resolution: 2.60 Å) [13], and the periplasmic binding protein FhuD (PDB ID: 1K2V, resolution: 1.97 Å) [15] were retrieved from the Protein Data Bank. Of these three proteins, FhuE was selected as the target for the molecular docking simulations, as detailed below, while FhuA and FhuD were prepared as structurally and functionally correlated members of the same ferric hydroxamate uptake pathway to provide structural context for the pathway-level discussion; they were not subjected to docking in the present study.

The crystallographic structures were imported into the SAMSON v.2023 environment (Software for Adaptive Modeling and Simulation Of Nanosystems) [1], where protein preparation was performed according to the following steps: (i) addition of hydrogen atoms according to the physiological state (pH = 7.4), using SAMSON’s internal protonation modules based on quantum chemistry and molecular mechanics parameters [2]; (ii) assignment of protonation and tautomeric states of ionizable residues, taking into account physiological conditions and the microenvironment of the binding site; (iii) selective removal of non-structural water molecules, retaining only those involved in relevant interactions with the co-crystallized ligand and active site residues, based on geometric and energetic criteria; (iv) structural optimization and energetic relaxation through energy minimization using the OpenMM Wizard extension [3] with the AMBER ff14SB force field [4] and TIP3P water model [5], ensuring the correction of local geometrics and resolution of steric clashes without compromising the overall crystallographic conformation.

#### 2.5.3. Binding Site Definition and Grid Generation for FhuE

For the receptors FhuE (PDB ID: 6E4V), active site definition did not require cavity prediction algorithms, since the selected crystallographic structures were in the holo conformation (complexed with their natural ligands). Therefore, the region of interest for molecular docking was based on the biological location of the co-crystallized siderophores.

The coordinates and dimensions of the three-dimensional search space (grid box) were defined for the FhuE receptor within the SAMSON v.2023 environment using the AutoDock Vina v1.2.5 extension. The grid center was positioned at the geometric centroid of the co-crystallized ligand of FhuE [13], and the box dimensions were manually adjusted to encompass all relevant active site residues. This setup ensured a sufficient search volume to explore the conformational space of the docked ligand without structural restrictions, while maintaining the focus on the established binding pocket. The specific grid center coordinates and box dimensions are reported in Section 2.5.5.

#### 2.5.4. Ligand Preparation: Structural Construction and QM Optimization of the Fe (III)–Deferoxamine Complex

The initial geometry of the Fe(III)–deferoxamine (Fe–DFO) complex was constructed within the Maestro workspace v.9.3 [1]. The molecular structure of deferoxamine was obtained from the PubChem database (SDF format) [2], ensuring the precise assembly of the polyamine chain and the three hydroxamate units. To accommodate ferric ion coordination, the hydroxamate groups were adjusted to their deprotonated form (O^−^). The Fe(III) ion was explicitly inserted, and the Fe–O coordination bonds were established with initial distances adjusted between 1.9 and 2.1 Å, consistent with an octahedral coordination sphere.

To eliminate steric clashes from manual construction, a preliminary minimization was performed using the MacroModel module with the OPLS4 force field (100–500 steps) [3]. The system’s total charge and spin multiplicity were assigned considering Fe(III) as a d^5^ high-spin system; independent optimizations were conducted for high-spin (multiplicity 6) and low-spin (multiplicity 2) states to identify the most stable electronic configuration.

**Figure 1:**
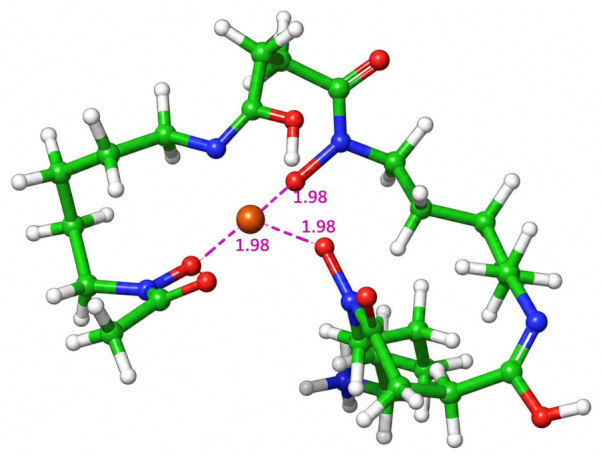
Optimized geometry of the Fe(III)–deferoxamine (Fe–DFO) complex. Atoms are colored by element according to the standard CPK scheme: carbon (green), oxygen (red), nitrogen (blue), and hydrogen (white). The central orange sphere represents the ferric ion, Fe(III). Dashed magenta lines indicate the coordination bonds between Fe(III) and the hydroxamate oxygen donors, with interatomic distances given in ångströms (Å). The three Fe–O coordination distances of 1.98 Å are consistent with the octahedral geometry characteristic of hexadentate siderophore–iron complexes.

The refined structures were then subjected to full quantum geometry optimization using the Jaguar program [4] (Figure 1). Calculations were performed within the framework of Density Functional Theory (DFT), employing the hybrid B3LYP functional [5] with Grimme’s empirical dispersion correction (D3) [6]. The def2-TZVP basis set was applied to all atoms to ensure a high-level description of the metal–ligand interface [7]. Self-consistent field (SCF) convergence was set to 1 × 10^−5^ Hartree. To simulate physiological environments, an implicit solvation model of the Poisson–Boltzmann finite element type (PBF) was employed with water as the solvent [8].

Following optimization, a single-point calculation was performed to evaluate the molecular electrostatic potential (ESP) [9]. The partial charges derived from the ESP fit were utilized to map the electronic distribution of the complex. The final quantum mechanical geometries, with strictly preserved metal coordination, were exported to the SAMSON v.2023 environment [10].

For the molecular docking stage, the structures were converted to the PDBQT format. To ensure compatibility with the AutoDock Vina scoring engine [13] while maintaining the high-level electronic information from DFT, the ESP-derived charges were mapped onto the atoms, with Gasteiger charges providing complementary refinement for the organic framework. Additionally, the protonation states of all ionizable groups in the ligand and the target protein (FhuE) were adjusted to pH 7.4 using the PROPKA algorithm [11]. This integrated workflow ensured that the refined quantum geometry and the physiological ionization states were maintained throughout the docking simulations, addressing the specific field-of-force requirements for metallic complexes.

#### 2.5.5. Molecular Docking Execution and Protocol Validation

Following the preparation of the receptors and the quantum-optimized Fe(III)–DFO complex, molecular docking simulations were performed within the SAMSON v.2023 environment using the AutoDock Vina extension. To account for receptor plasticity, the docking was conducted using a flexible receptor model, where specific residues in the binding pocket of FhuE (PDB ID: 6E4V) were defined in their flexible states, as previously prepared in Section 2.5.2.

The docking parameters were strictly defined to ensure high reproducibility and sampling depth. The search space was centered at coordinates X: 31.3, Y: 7.4, Z: 22.7, with grid box dimensions of 29.0 × 32.2 × 30.2 Å and a grid spacing of 0.375 Å. An exhaustiveness value of 16 was employed to ensure a robust exploration of the ligand’s conformational and orientational degrees of freedom.

To validate the docking protocol, a redocking procedure was performed using the co-crystallized ligand of the 6E4V structure. The top-ranked pose (Pose 1) achieved a binding affinity of −9.29 kcal/mol and a Root Mean Square Deviation (RMSD) of 0.4 Å when compared to the original crystallographic conformation. This low RMSD value, significantly below the 2.0 Å threshold, confirms the high accuracy of the docking parameters in replicating the experimental binding mode.

For the Fe(III)–DFO complex, 20 distinct poses were generated and ranked according to the Vina semi-empirical scoring function. The most energetically favorable conformation, exhibiting a binding free energy of −7.4 kcal/mol—a value consistent with weak-to-moderate structural fit within the scoring framework of AutoDock Vina, and not predictive of biological inhibitory activity in isolation—was selected for further post-docking analysis. This included a detailed visual inspection of intermolecular interactions to ensure structural consistency with the biological data described for the FhuE-mediated transport system.

### 2.6. In Vitro Antimicrobial Activity of DFO

#### 2.6.1. Minimum Bactericidal Concentration (MBC)

The Minimum Bactericidal Concentration (MBC) was determined from the serial microdilution wells corresponding to the Minimum Inhibitory Concentration (MIC) and higher concentrations. Aliquots from these wells were subjected to serial decimal dilutions and subsequently plated on solid medium free of the test compound. After incubation for 24 h at 37 °C, colony-forming units were counted, expressed as CFU/mL, and converted to log_10_. The reduction in bacterial viability was calculated as the difference between the log_10_ CFU/mL of the growth control and the log_10_ CFU/mL of the treated samples. The MBC was defined as the lowest concentration capable of reducing bacterial recovery below the detection limit of the plating method under the experimental conditions evaluated. Colony counts obtained from the serial dilutions were used to estimate the reduction in bacterial recovery in the treated samples relative to the growth control, using GraphPad Prism 8 software.

#### 2.6.2. Cytotoxicity Assay (RAW 264.7 Cell Line)

Murine RAW 264.7 macrophages (donated by the Applied Immunology Laboratory, Rio de Janeiro) were used for the cytotoxicity assays. Cells were maintained in sterile culture flasks with RPMI 1640 medium (SIGMA) containing penicillin (100 µg/mL), streptomycin (100 U/mL), and amphotericin B (0.25 µg/mL), supplemented with 5% fetal bovine serum (FBS), in an incubator at 5% CO_2_ and 37 °C.

For the cytotoxicity assay, 2 × 10^6^ cells/mL were used in RPMI-1640 medium supplemented with 1% FBS. Cells were seeded in a volume of 100 µL per well in flat-bottom 96-well plates. The macrophages were then incubated for 1 h in a 5% CO_2_ incubator to allow adherence to the plate bottom.

After this period, the macrophages were incubated with different concentrations of DFO (78.1 to 5000 µg/mL) for subsequent calculation of the CC50. As controls, wells with untreated cells (negative control) and cells treated with dimethyl sulfoxide (DMSO) (positive control) were maintained. After incubation for 48 h, MTT (3-(4,5-dimethylthiazol-2-yl)-2,5-diphenyltetrazolium bromide) (Sigma-Aldrich, St. Louis, MO, USA) (5 mg/mL) was added to the culture, followed by a further incubation (3 h at 37 °C).

Cell viability was assessed based on MTT metabolism, which is proportional to the absorbance generated in a spectrophotometer at a wavelength of 540 nm. After the 3 h incubation, 100 µL of DMSO was added per well and read in an ELISA reader (Thermo Fisher Scientific, Vantaa, Finland) at 540 nm. The percentage of cytotoxicity was calculated according to the following formula: % cytotoxicity = (1 − [optical density of test/mean of negative control]) × 100. Cytotoxicity was expressed as a percentage, and the 50% cytotoxic concentration was determined using GraphPad Prism 8 software (CC_50_).

## 3. Results

### 3.1. Cystitis in an Experimental Model

Induction of spinal cord injury resulted in immediate loss of locomotor function of the animals’ pelvic limbs, as well as complete loss of bladder function. After 24 h, a total inability to spontaneously empty the bladder was observed, characterized by the accumulation of large volumes of urine and the need for manual expression, indicating detrusor areflexia. This condition was confirmed by a significant increase in post-void residual volume during physical examinations.

Microbiological analysis of the collected urine samples revealed significant bacterial growth in all analyzed samples. Urine culture showed bacterial counts compatible with urinary tract infection, adopting values equal to or greater than 10^5^ colony-forming units per milliliter (CFU/mL) as the criterion for positivity. These findings confirm that the urinary stasis induced by the model favored bacterial colonization of the bladder. The isolated bacteria were identified as *E. coli* using the microbiological and biochemical methods described above.

Urinalysis revealed reddish-colored urine with a characteristic odor and turbid appearance, with the presence of visible deposits. Urine specific gravity was 1.052, a value above the commonly cited reference range for healthy freely voiding Wistar rats (1.022 to 1.050). This elevation is biologically consistent with the pathophysiological context of the model: following complete detrusor areflexia, urine was retained in the bladder for approximately 24 h without voiding, during which continued transepithelial water reabsorption by the bladder mucosa progressively concentrated the retained urinary content. Additionally, the active inflammatory/infectious process, evidenced by intense hematuria (++++), marked leukocyturia, and heavy bacteriuria, contributes particulate and macromolecular content that further elevates measured specific gravity beyond osmolar concentration alone. This finding is therefore an expected consequence of urinary stasis and concurrent infection rather than a measurement artifact. Urinary pH was 5.5, characterizing slightly acidic urine in relation to laboratory reference values. In the chemical evaluation, the absence of proteins, bilirubin, glucose, ketones, and urobilinogen was observed. However, a marked presence of hemoglobin (++++) and leukocytes (+++) and nitrite (+++) were detected. These findings indicate a condition compatible with an active inflammatory–infectious process of the urinary tract, accompanied by mucosal bleeding, characterizing hematuria associated with urinary tract infection. This profile is suggestive of infectious cystitis of moderate to high severity.

Analysis of the urinary sediment showed 15 to 20 leukocytes per field (40×) and 25 to 35 erythrocytes per field (40×), both above reference values, confirming an inflammatory process and bleeding in the urinary tract. Rare epithelial cells (bladder origin), absence of casts, and frequent presence of triple phosphate crystals were observed, as well as rare mucus filaments, in addition to the presence of bacteria in the sediment.

### 3.2. Docking Protocol Validation and Structural Analysis of FhuE Binding Modes

**Figure 2:**
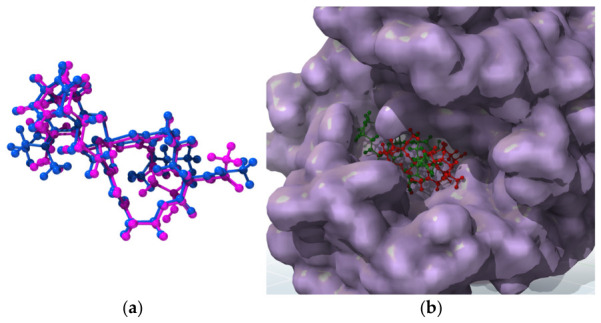
(**a**) Redocking validation: superposition between the native conformation of the co-crystallized ligand (pink) and the pose obtained after docking (blue). The RMSD of 0.4 Å confirms the accuracy of the search parameters and the reliability of the defined active site. (**b**) Binding mode prediction: representation of the deferoxamine–Fe^3+^ complex (green) superimposed on the reference co-crystallized ligand (red) within the active site. The target protein is displayed as a molecular surface, highlighting the geometric complementarity and the occupation of the relevant biological cavity by the Fe–DFO complex.

All molecular docking analyses reported in this study were performed on the FhuE receptor (PDB ID: 6E4V), and all results described below refer to the FhuE–Fe(III)–DFO complex. The molecular docking protocol was validated through a redocking procedure using the co-crystallized ligand of the FhuE receptor (PDB ID: 6E4V). The simulation successfully reproduced the experimental binding mode with a Root Mean Square Deviation (RMSD) of 0.4 Å (Figure 2a). This value, being significantly below the 2.0 Å threshold, confirms the geometric accuracy of the selected parameters—including grid dimensions and an exhaustiveness of 16—in exploring the relevant conformational space of the binding pocket (Figure 2a).

The calculated binding affinities were −9.29 kcal/mol for the reference ligand and −7.4 kcal/mol for the Fe(III)–DFO complex. In alignment with the limitations of docking scoring functions, these values are treated as qualitative indicators of structural complementarity rather than absolute thermodynamic constants. The higher affinity of the native ligand is consistent with its experimental origin, while the score for Fe(III)–DFO suggests that, despite its high polarity and flexibility, the complex presents a geometrically plausible orientation within the analyzed pocket (Figure 2a,b).

Analysis of the generated poses for Fe(III)–DFO revealed a predominant binding mode corresponding to the lowest-energy conformation. While secondary poses were identified with an internal RMSD variation of 2.5 Å, the energy gap between them (0.5 kcal/mol) falls within the inherent noise range of semi-empirical scoring functions. Consequently, these alternative orientations are interpreted as a manifestation of the ligand’s conformational flexibility within the large FhuE vestibule, rather than distinct functional binding sites. This approach ensures a cautious interpretation of the results, focusing on the structural plausibility of the primary pose which maintains the coordination sphere of the ferric ion in a manner consistent with the receptor’s architecture. For clarity: (i) no alternative binding site is proposed or validated in this study; (ii) the 0.5 kcal/mol inter-pose energy gap does not meet any established threshold for distinguishing biologically distinct binding modes; and (iii) all structural and mechanistic discussion in subsequent sections refers exclusively to the primary lowest-energy pose.

#### Intermolecular Interactions Within the FhuE Binding Site

**Figure 3:**
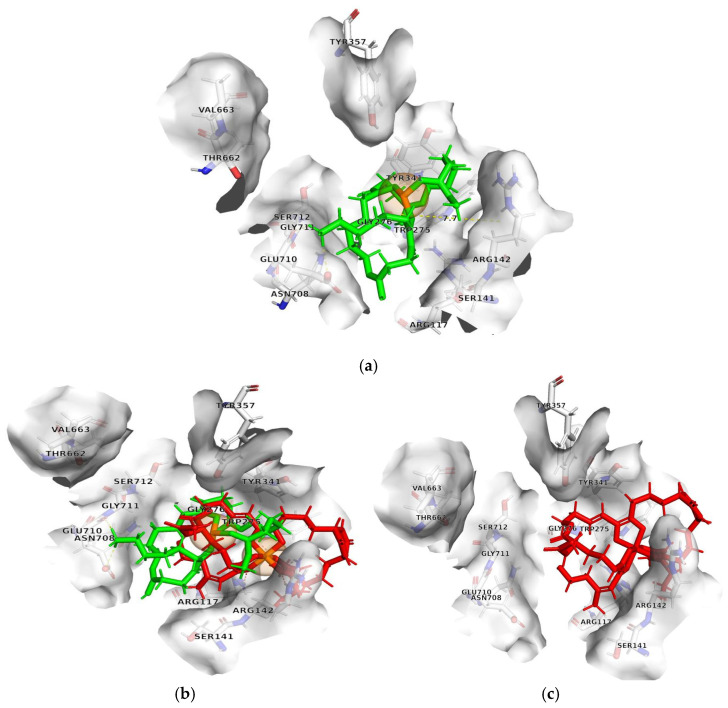
(**a**) Primary binding orientation: Atomic-level detail of the Fe–DFO complex in the main pocket, highlighting the directional hydrogen bond established with GLU710 (dashed line). A measurement tool indicates a spatial gap of 7.7 Å between the complex and ARG142, confirming the absence of classical hydrogen bonding at this position. Surrounding residues (ARG117, SER141, GLY276, TRP275, and TYR341) contribute primarily through steric fit and van der Waals contacts. (**b**) Native recognition site: Detail of the co-crystallized reference ligand, highlighting the established anchor interaction with ARG142 (dashed line), which serves as a polar stabilizer for the native siderophore. (**c**) Structural superposition: Overlay of the docked Fe–DFO complex (red) and the co-crystallized ligand (green/blue), demonstrating spatial convergence within the central vestibule. The alignment confirms that both ligands occupy a comparable volume of the cavity, supporting the qualitative plausibility of the predicted binding mode despite the divergence in specific directional contacts.

Detailed structural analysis of the predicted binding modes for the Fe–DFO complex within the FhuE receptor reveals a pattern of steric accommodation consistent with the pocket topology, supported by a limited number of directional polar interactions. As observed in the structural representations (Figure 3a), the only clearly identifiable hydrogen bond for Fe–DFO occurs with the GLU710 residue. Other residues, such as ARG117, SER141, GLY276, and TRP275, provide a chemical environment where interactions are predominantly governed by steric fit or van der Waals contacts within the cavity, rather than established directional hydrogen bonds.

In contrast, the analysis of the co-crystallized ligand (Figure 3b) confirms a well-defined hydrogen bond with the ARG142 residue, which serves as a key polar anchor for the native siderophore. Under the specific docking conditions for the Fe–DFO complex, however, the distance between the ferric center and the ARG142 side chain was measured at 7.7 Å. At this distance, no direct polar interaction or significant electrostatic contribution can be inferred. This spatial gap suggests that, while the Fe–DFO complex is accommodated within the same general vestibule as the native ligand, it does not replicate the specific recognition pattern involving ARG142. Instead, its localization appears to be governed by a distinct set of steric constraints and the single hydrogen bond identified with GLU710.

To further evaluate the plausibility of the predicted binding mode, a structural superposition between the docked Fe–DFO complex and the co-crystallized reference ligand was performed (Figure 3c). The overlay reveals a substantial spatial convergence within the central binding vestibule of FhuE, particularly in the region defined by TRP275, GLY276, and ARG142. Both ligands occupy a comparable volume of the cavity, supporting the notion that the docking protocol correctly positioned Fe–DFO within the biologically relevant recognition site. Despite this overall spatial overlap, subtle conformational differences are evident; whereas the native ligand establishes a defined contact with ARG142, Fe–DFO engages the same functional vestibule through a partially distinct recognition strategy, relying on alternative anchoring patterns and geometric complementarity.

Additional contacts based on geometric proximity, including those involving TYR341 and TRP275, were interpreted with caution as distance-based geometric fits rather than definitive stabilizing interactions. The presence of a single direct hydrogen bond suggests that Fe–DFO stabilization within the binding site relies primarily on geometric complementarity and diffuse non-covalent forces, consistent with the highly polar nature of deferoxamine. Alternative orientations involving residues such as ASN708, GLY711, and SER712 were also identified but are treated as indicators of structural plasticity and conformational flexibility within the large FhuE vestibule.

### 3.3. In Vitro Assays

#### 3.3.1. Minimum Bactericidal Concentration (MBC)

**Figure 4:**
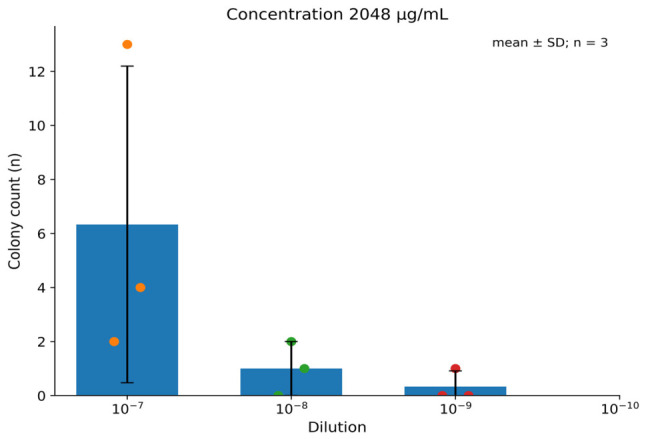
Colony-forming units of *E. coli* quantified after serial dilution and plating with a 1 µL loop at dilutions 10^−8^, 10^−9^, and 10^−10^ following exposure to DFO at 2048 µg/mL. Bars represent mean colony counts from three replicates; individual values are shown as points. Percentage reductions relative to the growth control: 61.2% at 10^−8^ and 66.7% at 10^−9^, with absence of growth at 10^−10^.

Colony-forming units were quantified after serial dilution and plating with a 1 µL loop. Figure 4 presents the number of colonies observed at dilutions 10^−8^, 10^−9^, and 10^−10^. A progressive reduction in colony counts was observed across the dilution series, indicating decreased bacterial recovery at higher dilutions.

The bactericidal activity of DFO demonstrated a marked reduction in bacterial recovery at a concentration of 2048 µg/mL. After serial dilution and plating with a 1 µL loop, a progressive decrease in colony counts was observed, with approximately 4 colonies at 10^−8^, 1 colony at 10^−9^, and no growth at 10^−10^. Relative to the growth control, these findings corresponded to a reduction of approximately 61.2% at 10^−8^ and 66.7% at 10^−9^. The presumptive MBC of DFO was established at 2048 µg/mL, the concentration at which bacterial reduction was observed with absence of growth at the 10^−10^ dilution. Although residual colonies were observed at 10^−8^ and 10^−9^, the overall findings indicate that 2048 µg/mL represented the highest concentration tested and the concentration at which colony recovery fell below the detection limit at the highest dilution. DFO thus demonstrated a concentration-dependent reducing effect on the recovery of *E. coli*; a precise log_10_ reduction was not quantified, as this would require absolute CFU/mL determination from direct plating of undiluted or minimally diluted treated cultures against the initial inoculum.

**Figure 5:**
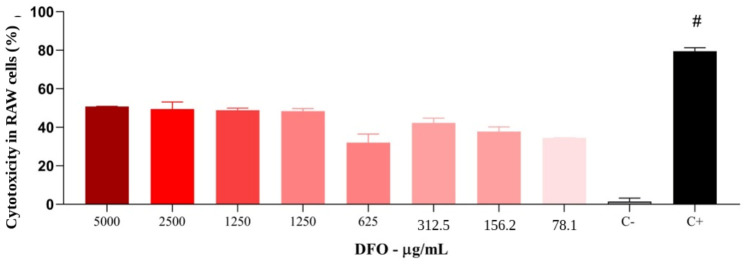
Cytotoxicity profile of deferoxamine (DFO) in RAW 264.7 murine macrophages. Cytotoxicity was evaluated across a concentration range of 78.1 to 5000 µg/mL (95% confidence interval shown). Results correspond to means ± SD of individual samples tested in triplicate. (#) *p* < 0.05 compared to the positive control (DMSO).

The cytotoxicity profile of deferoxamine (DFO) in RAW 264.7 cells is presented in Figure 5. A moderate, dose-dependent toxicity profile was observed across the tested concentrations. Although DFO induced a reduction in cell viability, the cytotoxicity percentage did not substantially exceed the 50% threshold even at the maximum concentration, suggesting relative tolerance of RAW cells to the compound at lower doses.

#### 3.3.2. Cytotoxic Concentration CC_50_

**Figure 6:**
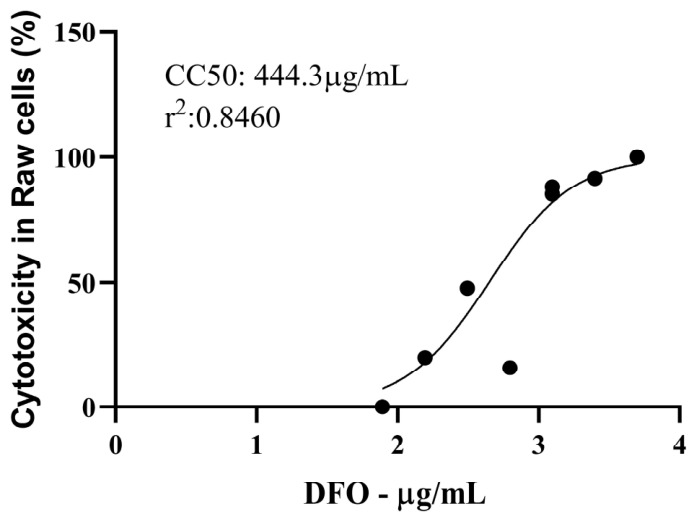
Dose–response curve of DFO in RAW 264.7 murine macrophages. CC50 = 444.3 µg/mL.

In order to determine the pharmacological safety of the compound, the 50% cytotoxic concentration was calculated (CC_50_), which represents the concentration required to reduce cell viability by 50% relative to the untreated control (Figure 6).

The calculated CC_50_ value is considerably higher than the lowest tested doses, allowing the selection of safe concentrations for subsequent biological activity assays of DFO, preferentially those below the 62.5 µg/mL threshold, at which cytotoxicity was minimized.

DFO presented a CC50 of 444.3 µg/mL in RAW 264.7 cells, suggesting moderate cytotoxicity and a relatively tolerable profile at lower concentrations.

## 4. Discussion

The molecular docking results obtained in this study provide a structural basis for evaluating the qualitative plausibility of Fe–DFO accommodation within the FhuE receptor of *Escherichia coli*. Although the predicted binding affinity for the Fe–DFO complex (−7.4 kcal·mol^−1^) was lower than that of the native co-crystallized ligand (−9.29 kcal·mol^−1^), this discrepancy is expected when comparing an exogenous, highly flexible chelator to an evolutionarily optimized natural siderophore [6,11]. Within the context of docking scoring functions, these values should be interpreted not as absolute thermodynamic constants, but as indicators of structural complementarity [13].

A key finding of this study is the spatial convergence observed between Fe–DFO and the reference ligand. As demonstrated by the structural superposition (Figure 3c), both molecules occupy a comparable volume within the central binding vestibule of FhuE. This overlap suggests that the docking protocol correctly identified the biologically relevant recognition site [13,14], despite the high polarity and many degrees of freedom of the deferoxamine framework. However, the specific interaction patterns reveal a clear divergence in recognition strategies. While the native ligand relies on a well-defined polar anchor at ARG142, the Fe–DFO complex maintains a significant spatial gap of 7.7 Å from this residue. At such a distance, direct polar interactions or significant electrostatic contributions are unlikely, indicating that Fe–DFO does not replicate the precise anchoring mechanism of the native siderophore.

Instead, the stabilization of Fe–DFO appears to be governed by a single directional hydrogen bond with GLU710 and a broader network of non-specific steric and van der Waals contacts with residues such as TRP275 and TYR341. This “loose” binding mode is consistent with the structural plasticity required for exogenous chelators to be accommodated by bacterial transporters [6,16]. By moving away from overinterpreting docking scores as evidence of “functional inhibition,” this analysis aligns with contemporary literature that views siderophore–receptor interactions as dynamic processes where geometric fit often precedes specific polar recognition [11,13]. The lack of Molecular Dynamics (MD) simulations is partially mitigated by the use of high-level DFT refinement for the ligand’s coordination sphere, ensuring that the predicted geometries are electronically accurate. While future studies may explore the temporal stability of these contacts, the current static model successfully establishes the qualitative feasibility of Fe–DFO being recognized by the FhuE transport system [13].

The broader context of siderophores and iron chelators in microbial biology has implications that go beyond simple molecular recognition. Iron acquisition via siderophores is a critical process for the pathogenicity of many bacteria and has been extensively studied as a potential therapeutic target [4,7,17]. Innovative strategies, such as artificial siderophores or conjugates that exploit bacterial transport pathways to deliver antimicrobial agents (e.g., “Trojan Horse” strategies), demonstrate the translational value of these interactions [6,11]. In addition, iron deprivation by chelating agents has shown antibiofilm effects and may potentiate the efficacy of antibiotics, although the results vary depending on the microorganism and the compound used [18,19].

It is important to delimit the interpretive scope of the present analysis. Iron acquisition in *E. coli* is mediated by multiple parallel systems, including the enterobactin pathway (FepA), the aerobactin system, catecholate transporters, and haem acquisition routes, in addition to the ferric hydroxamate uptake (Fhu) pathway examined here. The Fhu system was selected on three specific grounds. First, deferoxamine is a hydroxamate-type chelator, making FhuA, FhuE, and FhuD the chemically most plausible interaction partners, since these proteins evolved to recognize hydroxamate-coordinated ferric complexes; an exogenous hydroxamate is far less likely to engage catecholate or haem systems that recognize structurally distinct iron complexes. Second, high-resolution holo-conformation crystal structures with co-crystallized native ligands are available for all three Fhu targets, enabling experimentally grounded binding-site definition, whereas equivalent structural data for other *E. coli* iron-acquisition proteins are either unavailable or of lower resolution. Third, the hydroxamate-type pathway is active in uropathogenic strains under the iron-restrictive urinary conditions reproduced by the neurogenic bladder model. Accordingly, all interpretive statements in this work are confined to the Fhu-mediated ferric hydroxamate acquisition pathway and should not be extrapolated to global iron homeostasis. Future studies should extend this analysis to additional systems, particularly FepA-mediated enterobactin uptake, the predominant route in most uropathogenic *E. coli* isolates, and to haem acquisition systems potentially relevant in the haematuria-associated infection documented here.

Several methodological limitations of the computational approach should be acknowledged. The docking protocol was validated by redocking the co-crystallized native ligand, yielding an RMSD of 0.4 Å against the crystallographic reference, well below the 2.0 Å acceptance threshold; however, enrichment-based validation using known actives and decoys was not performed, and prospective applications of this docking setup to compound screening should include such discrimination tests. Molecular dynamics simulations and end-point free-energy recalculations (MM-PBSA/MM-GBSA) were likewise not performed; consequently, the predicted binding modes describe static geometric feasibility rather than dynamic binding stability or rigorous binding free energies. The absence of these analyses is partially mitigated by the high-level DFT refinement of the Fe–DFO coordination sphere, which ensures electronically accurate input geometry, but it does not substitute for dynamic or thermodynamic characterization. These analyses, together with the functional and in vivo experiments described above, constitute the priority directions for the computational and experimental continuation of this work.

In the present study, the isolation of *E. coli* in the neurogenic bladder model, evidenced by high leukocyte and nitrite counts, underscores the clinical relevance of studying iron acquisition targets [1,12,20]. This observation is consistent with prior characterization of urinary tract infection in the spinal cord-injured host with neurogenic bladder, in which host-dependent mechanisms beyond urinary stasis were shown to govern infection susceptibility [12]; such host–pathogen interaction nodes, including bacterial iron acquisition systems, represent rational targets that operate independently of bladder physiology. The docking data provide in silico support for the structural plausibility of Fe–DFO interactions, opening the way for hypotheses regarding interference with Fhu-mediated iron acquisition. We emphasize that the infection model and the computational analyses are presented here as complementary components of a proof-of-concept framework rather than as a fully integrated experimental system: the model provides the validated pathophysiological context and the biological isolate, while the docking generates structural hypotheses that remain to be tested by the functional and in vivo experiments identified as future priorities. However, it is crucial to emphasize that these interactions do not directly imply mechanisms of catalytic inhibition or protein blockade; rather, they point to a potential for structural binding that may, if experimentally confirmed, influence Fhu-mediated iron acquisition specifically, rather than iron-dependent metabolic pathways in general [21]. These results reinforce the idea that deferoxamine, while not a traditional antibiotic, possesses interactive properties that make it a candidate for future studies focused specifically on interference with the Fhu-mediated ferric hydroxamate pathway in Gram-negative pathogens, rather than on global iron homeostasis [19,21].

At the concentration of 2048 µg/mL, deferoxamine promoted a marked reduction in bacterial colony counts compared to the growth control. At the 10^−8^ dilution, approximately 4 colonies were observed in the treated sample, while the control presented a mean of approximately 10.3 colonies, corresponding to a reduction of approximately 61.2% in bacterial viability. Similarly, at the 10^−9^ dilution, DFO yielded only 1 colony compared to approximately 3 colonies in the control, representing a reduction of 66.7% relative to growth control. Furthermore, at the 10^−10^ dilution, no bacterial growth was observed in the treated sample, indicating a reduction in viable cell recovery below the detection limit of the plating method following compound exposure. Because the absence of growth at this dilution does not yield a quantifiable CFU/mL value for the treated sample, a specific log_10_ reduction cannot be established from these data alone. The results are therefore interpreted as evidence of a concentration-dependent reduction in bacterial recovery rather than as a quantified reduction in the *E. coli* population under the conditions evaluated. Taken together, these findings suggest that DFO exhibits measurable antibacterial activity under the conditions evaluated [6,11]. It should be noted, however, that the MBC of 2048 µg/mL exceeds concentrations typically associated with direct therapeutic applicability; this value is therefore interpreted as evidence of intrinsic antibacterial activity in a proof-of-concept context rather than as a directly translatable therapeutic dose. Functional assays under iron-restricted conditions, intracellular iron quantification, and biofilm evaluation are required to establish the specific contribution of iron acquisition interference to this effect and are identified as priorities for future work.

The cytotoxicity results demonstrated that DFO exhibited moderate, dose-dependent toxicity in RAW 264.7 cells, with an estimated CC50 of 444.3 µg/mL. It should be noted that the presumptive MBC of 2048 µg/mL substantially exceeds the CC50, by a factor of approximately 4.6; the concentration required for antibacterial activity under these experimental conditions would therefore be expected to be associated with considerable cytotoxicity in host cells. This represents an important limitation and underscores that the present study does not support a claim of therapeutic applicability at the tested concentrations. The biological activity data are accordingly interpreted solely as proof-of-concept evidence that DFO exerts measurable inhibitory effects on *E. coli* recovery under defined experimental conditions, rather than as evidence of therapeutic feasibility. Concentrations below 62.5 µg/mL, at which cytotoxicity was minimized, define the sub-toxic range relevant for future mechanistic assays; these concentrations are well below the tested MBC and are not themselves associated with the observed antibacterial effect. Given that DFO acts as an iron chelator, its evaluation in host cells remains relevant, since DFO modulates iron-dependent cellular processes in RAW 264.7 macrophages that may bear on the immunological response to infection [19].

## 5. Conclusions

In summary, the experimental model of neurogenic bladder induced by spinal cord transection proved to be effective in reproducing functional alterations compatible with severe bladder dysfunction, including persistent urinary retention and predisposition to urinary tract infection by *E. coli*. within this pathophysiological context, the in silico analyses provided support for the plausibility of interactions between the deferoxamine–Fe^3+^ complex and bacterial proteins, demonstrating geometrically plausible binding interactions involving key residues of the FhuE binding site. Although these interactions do not directly imply classical mechanisms of enzymatic inhibition, they suggest potential for structural association consistent with the hypothesis that structural binding of this nature may, if experimentally confirmed, influence iron-dependent metabolic pathways. Taken together, the energetic, structural, experimental, and computational findings are consistent with the contemporary literature on siderophores and bacterial iron acquisition and reinforce the concept that deferoxamine, although not a traditional antibiotic, possesses chemical and interactive properties that make it a structurally relevant candidate for hypothesis-driven future studies focused on interference with the Fhu-mediated ferric hydroxamate acquisition pathway as a potential adjuvant strategy in the control of bacterial infections associated with neurogenic bladder, particularly in Gram-negative pathogens such as *E. coli*.

## Data Availability

The original contributions presented in this study are included in the article. Further inquiries can be directed to the corresponding author.

## Abbreviations

The following abbreviations are used in this manuscript:

ABC	ATP-Binding Cassette
BBB	Basso, Beattie and Bresnahan scale
NB	Neurogenic Bladder
CFU	Colony-Forming Unit
DFO	Deferoxamine
DFT	Density Functional Theory
ESP	Electrostatic Potential
Fe–DFO	Iron(III)–Deferoxamine complex
Fe^3+^	Ferric ion
FhuA	Ferric hydroxamate uptake protein A
FhuD	Ferric hydroxamate-binding periplasmic protein D
FhuE	Ferric hydroxamate uptake protein E
UTI	Urinary Tract Infection
MIC	Minimum Inhibitory Concentration
OPLS4	Optimized Potentials for Liquid Simulations 4
PBF	Poisson–Boltzmann Finite Element
PDB	Protein Data Bank
RMSD	Root Mean Square Deviation
SCF	Self-Consistent Field
CC50	50% Cytotoxic Concentration
CEUA	Ethics Committee on Animal Use
DMSO	Dimethyl Sulfoxide
EMB	Eosin Methylene Blue
FBS	Fetal Bovine Serum
MBC	Minimum Bactericidal Concentration
MTT	3-(4,5-Dimethylthiazol-2-yl)-2,5-Diphenyltetrazolium Bromide
RBCs	Red Blood Cells
SBCAL	Brazilian Society of Laboratory Animal Science
TSI	Triple Sugar Iron
WBCs	White Blood Cells
